# MicroRNA-27a-3p Inhibits Melanogenesis in Mouse Skin Melanocytes by Targeting Wnt3a

**DOI:** 10.3390/ijms160510921

**Published:** 2015-05-14

**Authors:** Yuanyuan Zhao, Pengchao Wang, Jinzhu Meng, Yuankai Ji, Dongmei Xu, Tianzhi Chen, Ruiwen Fan, Xiuju Yu, Jianbo Yao, Changsheng Dong

**Affiliations:** 1 College of Animal Science and Technology, Shanxi Agricultural University, Taigu 030801, China; E-Mails: 84840293@163.com (Y.Z.); wangsh0402@163.com (P.W.); mjz122021@126.com (J.M.); codecass@163.com (Y.J.); carrol-xv@sohu.com (D.X.); chentianzhi15@163.com (T.C.); ruiwenfan@163.com (R.F.); yxjfkh@126.com (X.Y.); 2 Laboratory of Animal Biotechnology and Genomics, Division of Animal and Nutritional Sciences, West Virginia University, Morgantown, WV 26506, USA; E-Mail: Jianbo.Yao@mail.wvu.edu

**Keywords:** melanogenesis, Wnt3a, miR-27a-3p, post-transcriptional regulation

## Abstract

MicroRNAs (miRNAs) play an essential role in the regulation of almost all the biological processes, including melanogenesis. MiR-27a-3p is nearly six times higher in white alpaca skin compared to brown skin, which indicates that miR-27a-3p may be a candidate regulator for melanogenesis. Wnt3a plays an important role in promoting melanoblasts to differentiate into melanocytes and melanogenesis. To confirm the function of miR-27a-3p to melanogenesis in mammals, miR-27a-3p mimic, inhibitor and their negative control were transfected into mouse melanocytes. As a result, miR-27a-3p inhibits melanogenesis by repressing Wnt3a at post-transcriptional level. A significant decrease in Wnt3a luciferase activity was observed in 293T cells co-transfected with the matched luciferase reporter vector and pre-miR-27a. Furthermore, the presence of exogenous miR-27a-3p significantly decreased Wnt3a protein expression rather than mRNA and reduced β-catenin mRNA levels in melanocytes. The over-expression of miR-27a-3p significantly increased the melanin content of melanocytes. However, miR-27a-3p inhibitor performs an opposite effect on melanogenesis. Wnt3a is one target of miR-27a-3p. MiR-27a-3p could inhibit Wnt3a protein amount by post-transcriptional regulation and melanogenesis in mouse melanocytes. Previous studies reported that Wnt3a promoted melanogenensis in mouse melanocytes. Thus, miR-27-3p inhibits melanogenesis by repressing Wnt3a protein expression.

## 1. Introduction

Coat color is determined mainly by the synthesis and distribution of melanin, which is synthesized in melanosomes of melanocytes and transferred to the adjacent keratinocytes, where melanins are accumulated to generate pigmented skin or hairs [1,2,3]. Wnts play important roles in cell fate, proliferation, differentiation and migration by activating receptor-mediated signaling pathways [4,5,6,7]. Wnt3a can specify neural crest cells to become melanocytes [8,9] and act on melanoblasts to maintain Mitf expression and promote melanoblasts to differentiate into melanocytes [8].

MicroRNAs (miRNAs) are highly conserved, single-stranded noncoding short RNA molecules (18–24 nucleotides) that regulate gene expression at the posttranscriptional level. Studies on the functions of miRNAs in melanogenesis are limited, although many miRNAs were shown to play a significant role in melanoma [10,11]. Over-expression of miR-25 in melanocytes reduced Mitf mRNA and protein abundance [2]. Over-expression of miR-137 in C57 mouse resulted in the production of mice with brown and gray skin colors [12]. Other miRNAs known to be involved in the pigmentation process include miR-340 and miR-145 [13,14].

Based on the skin miRNAomes of alpaca with brown and white coat color, expression of miR-27a-3p is nearly six times higher in white alpaca skin compared to brown skin [15]. In this study, we provided evidence to support a functional role of miR-27a-3p in inhibiting melanogenesis in mouse melanocytes by repressing Wnt3a.

## 2. Results and Discussion

### 2.1. Expression of MiR-27a-3p and Wnt3a mRNA in Brown and Gray Mouse Skin

**Figure 1:**
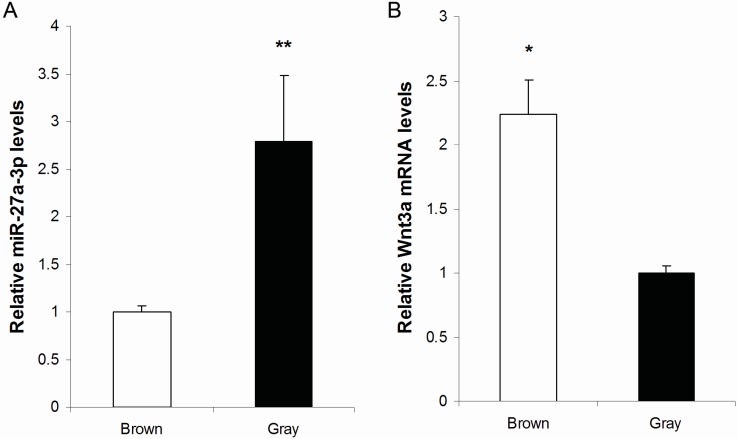
Expression of miR-27a-3p and Wnt3a mRNA in brown and gray mouse skin. (**A**) The relative expression of miR-27a-3p in gray mouse skin is significantly higher than that in brown mouse skin; (**B**) However, the expression of Wnt3a in brown and gray skin shows a contrary trend. Values represent the mean ± SE from four independent experiments. * *p* < 0.05, ** *p* < 0.01.

MiR-27a-3p was shown to be expressed significantly higher in white skin compared to brown skin of alpaca [15]. We performed real time PCR analysis to determine the expression of miR-27a-3p in gray *vs.* brown mouse skin. Results indicated that the relative expression of miR-27a-3p in gray mouse skin is significantly higher (3.14 times, *p* < 0.01) than that in brown mouse skin (Figure 1A). Real time PCR analysis revealed that the expression of Wnt3a mRNA in brown skin is significantly higher compared to gray skin (*p* < 0.05) (Figure 1B). The inverse relationship between the expressions of miR-27a-3p and Wnt3a mRNA in brown *vs.* gray mouse skin suggests that Wnt3a might be a potential target of miR-27a-3p.

### 2.2. MiR-27a-3p Targets the Predicted miRNA Binding Site in the 3' UTR of Wnt3a

**Figure 2:**
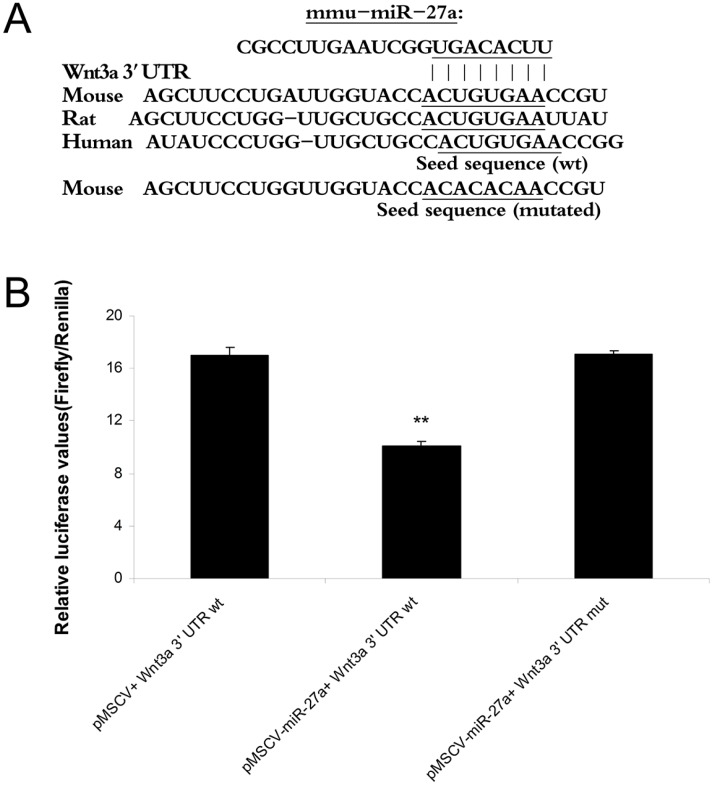
Wnt3a is a target of miR-27a-3p. (**A**) MiR-27a-3p binding site on the Wnt3a 3' UTR is shown, together with its homology across species; (**B**) Luciferase normalized to Renilla activity was measured in homogenates of HEK293T cells transfected with luciferase constructs containing the wild-type (wt) or mutated (mut) seed sequences of miR-27a-3p together with pMSCV-pre-miR-27a, or control pMSCV. Measurements were done 48 hours after transfection. The luciferase activity of cells co-transfected pGL0 Wnt3a 3' UTR (wt) with pMSCV-pre-miR-27a decreased 41%. ** *p* < 0.01.

The target genes of miR-27a-3p were predicted using miRanda (http://www.microRNA.org/), TargetScan (http://www.targetscan.org/) and DIANA-microT (http://www.microrna.gr/microT). One of the consensus target genes related to melanogenesis was Wnt3a (Figure 2A). Mouse Wnt3a cDNA is 2791 bp in length (GenBank accession number: NM_009522) and has a relatively long 3' UTR (1629 bp). The predicted binding site for miR-27a-3p lies between positions 2457 to 2464 bp in Wnt3a cDNA. To validate the specificity of miR-27a-3p regulation of Wnt3a through the predicted miRNA binding site, luciferase reporter assays were performed using luciferase reporter constructs containing either the wild type Wnt3a 3' UTR (pmirGL0-Wnt3a-wt-3' UTR) or the mutant Wnt3a 3' UTR (pmirGL0-Wnt3a-mut-3' UTR). The constructs were co-transfected into HEK293T cells with the pMSCV-miR-27a-3p expression plasmid or negative control plasmid. Compared with the cells transfected with pmirGL0-Wnt3a-wt-3' UTR and the negative control plasmid, the luciferase activity in cells co-transfected with pMSCV-miR-27a-3p and pmirGL0-Wnt3a-wt-3' UTR was decreased by 41%, while the luciferase activity in cells co-transfected with pMSCV-miR-27a-3p and pmirGL0-Wnt3a-mut-3' UTR was not decreased (Figure 2B). The results indicate that miR-27a-3p can bind and regulate Wnt3a specifically through the predicted binding site in the 3' UTR of the gene.

### 2.3. Over-Expression of MiR-27a-3p in Melanocytes Inhibits the Expression of Wnt3a and β-Catenin

**Figure 3:**
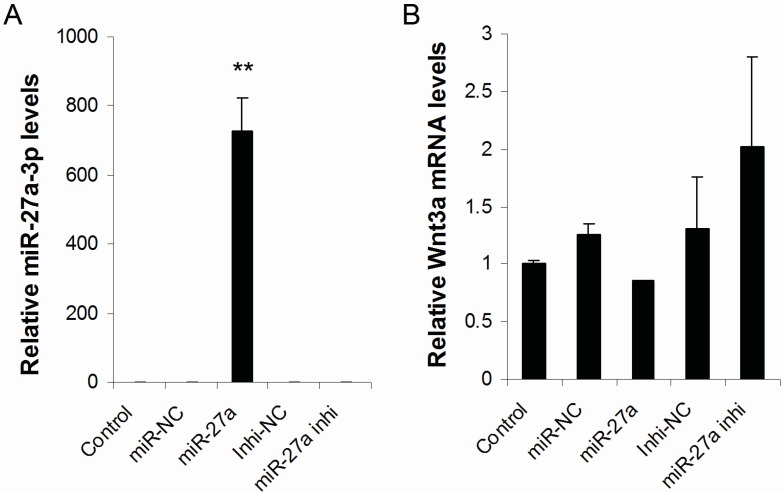
Expression levels of miR-27a-3p and Wnt3a mRNA in miR-NC, miR-27a, Inhi-NC, miR-27a inhi and Control group. (**A**) The miR-27a-3p expression level (728.6) is significantly higher in miR-27a group compared to other groups (Control: 1, Inhi-NC: 0.72, miR-27a inhi: 0.23, miR-NC: 1.92) (*p* < 0.01). The expression levels of miR-27a-3p in miR-27a inhi groups are significantly less than other groups (*p* < 0.05); (**B**) There are no significant difference in Wnt3a mRNA expression levels between control and other groups. Values represent the mean ± SE from four independent experiments. ** *p* < 0.01.

To evaluate the effect of miR-27a-3p on the expression of endogenous Wnt3a in melanocytes, Wnt3a protein was stained using immunofluorescence in mouse melanocytes and cultured mouse melanocytes were transfected with miR-27a-3p mimic or inhibitor, and their negative controls. The result shows that Wnt3a protein is expressed in mouse melanocytes, mostly around the cell nucleus (data not shown). The treatment groups included control (untransfected), miR-NC (miR-27a-3p mimic negative control), miR-27a (miR-27a-3p mimic), inhi-NC (miR-27a-3p inhibitor negative control), and miR-27a inhi (miR-27a-3p inhibitor). Real time PCR analysis shows that expression of miR-27a-3p is significantly higher in miR-27a mimic transfected group compared to other groups (*p* < 0.01). The expression of miR-27a-3p in miR-27a inhi group is significantly less than other groups (*p* < 0.05) (Figure 3A). The results indicate that miR-27a-3p mimic, inhibitor and their NC can be transfected into melanocytes efficiently.

**Figure 4:**
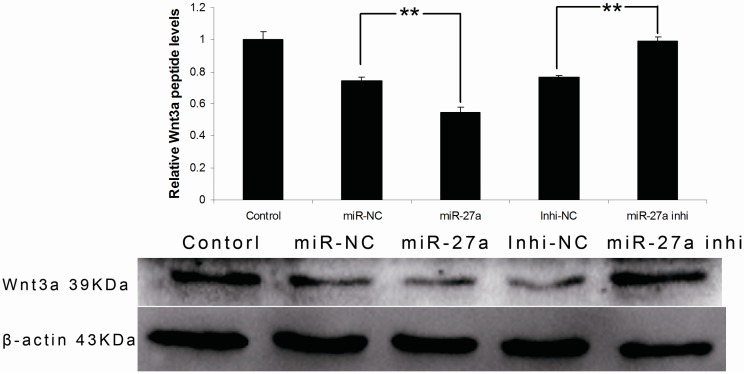
Expression levels of Wnt3a protein in melanocytes transfected with miR-27a mimic or inhibitor as well as their controls. Treated cells were divided into 5 groups: miR-NC, miR-27a, Inhi-NC, miR-27a inhi and untransfected control. Values represent the mean ± SE from four independent experiments. ** *p* < 0.01.

Expression of Wnt3a mRNA and protein in the miR-27a mimic or inhibitor-transfected groups were quantified by real-time PCR and Western blotting, respectively. The results indicated that there are no significant differences in Wnt3a mRNA expression among different groups (Figure 3B). However, the amounts of Wnt3a protein in various groups were significantly different. The level of Wnt3a protein was decreased in the miR-27a group compared with the miR-NC group (*p* < 0.01, Figure 4). In contrast to miR-27a-3p mimic treatment, miR-27a-3p inhibitor treatment displayed the opposite effect on Wnt3a protein expression: Wnt3a expression was increased in the miR-inhi group compared with that in the inhi-NC group (*p* < 0.01, Figure 4). There was no obvious difference between the miR-NC group and the inhi-NC group. Collectively, the results indicate that over expression of miR-27a-3p inhibits Wnt3a expression at protein level. These results demonstrate that miR-27a-3p can regulate Wnt3a expression by affecting Wnt3a protein translation. This agrees with the rules of miRNA actions on target genes [16,17].

**Figure 5:**
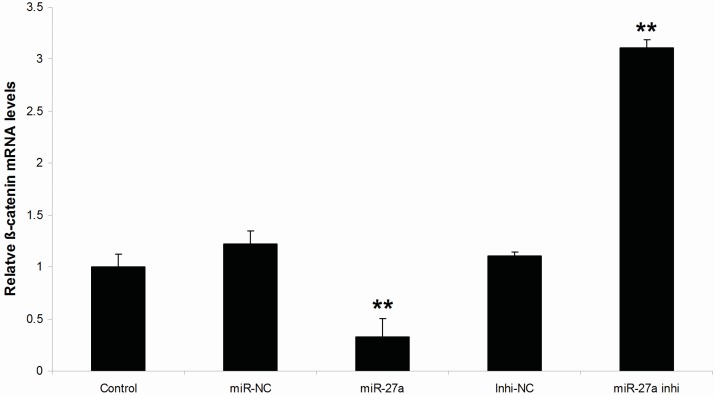
Expression levels of β-catenin mRNA in melanocytes transfected with miR-27a mimic or inhibitor as well as their controls. Treated cells were divided into 5 groups: miR-NC, miR-27a, Inhi-NC, miR-27a inhi and untransfected control. Values represent the mean ± SE from four independent experiments. ** *p* < 0.01.

Expression of β-catenin mRNA in the miR-27a mimic or inhibitor or their negative control transfected groups were determined by real-time PCR. The results showed that the β-catenin mRNA level in miR-27a group was significantly decreased compared with the miR-NC group (*p* < 0.01, Figure 5). On the contrary, the β-catenin mRNA level in miR-27a inhi group was markedly increased compared with in Inhi-NC group (Figure 5). Results indicate that miR-27a affects Wnt3a protein expression, which in turn affects β-catenin expression.

### 2.4. Over-Expression of MiR-27a-3p in Melanocytes Inhibits Melanogenesis

**Figure 6:**
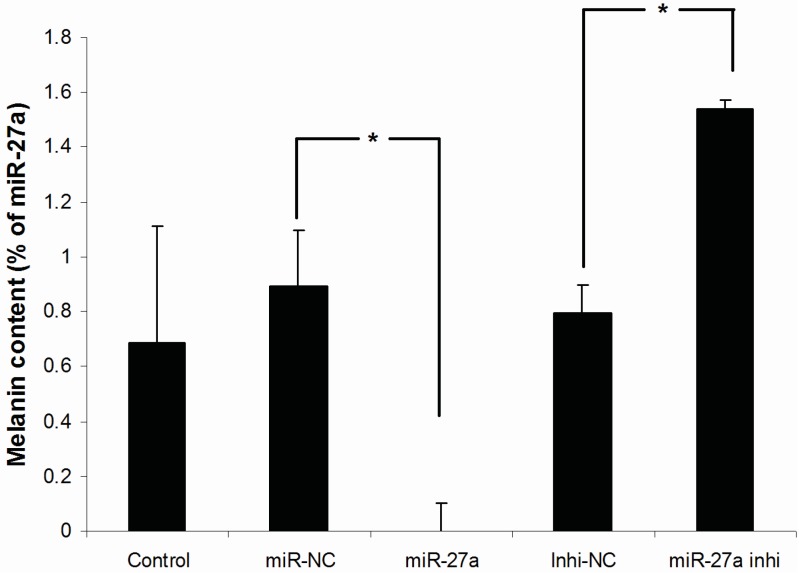
Melanin contents in melanocytes transfected with miR-27a mimic or inhibitor as well as their controls. Treated cells were divided into 5 groups: miR-NC, miR-27a, Inhi-NC, miR-27a inhi and untransfected control. MiR-27a groups are reference as 0. Values represent the mean ± SE from four independent experiments. * *p* < 0.05.

To determine the effect of miR-27-3p on the production of melanin in melanocytes, the melanin contents in the cells transfected with miR-27a-3p mimic or miR-27a-3p inhibitor, as well as their negative controls were measured. The results showed that the melanin content was decreased in the miR-27a group compared with the miR-NC group (*p* < 0.05, Figure 6). By contrast, the miR-27a inhi group displayed the opposite effect on melanin production to miR-27a group: the melanin content was increased compared with that of the inhi-NC (*p* < 0.05, Figure 6). There was no difference between the control group and the miR-NC group. Similarly, there was no difference between the control group and the inhi-NC group. These data suggest that over expression of miR-27a-3p in melanocytes could inhibit melanogenesis.

As a result, miR-27a-3p inhibits melanogenesis, Wnt3a protein expression and β-catenin in mouse melanocytes. Our results are in accord with previous study. In Wnt/β-catenin signaling pathway, Wnt ligands bind to Frizzled (Fzd) family of receptors and co-receptors, which leads to stabilization and translocation of β-catenin to the nucleus where it interacts with TCF/LEF family transcription factors to regulate the transcription of target genes [5]. Wnt3a proteins exert many of their effects by activating the expression of target genes through the stabilization and nuclear accumulation of β-catenin in mammalian cells [18]. Previous study have reported that Wnt3a could promote melanin accumulation, increase Tyr activity and the expression levels of Mitf, Tyr and Tyrp1 in melan-a cells [3]. In addition, Wnt3a induces the expression of endogenous Mitf-M mRNA by transactivating the Mitf-M promoter via the Lef-1-binding site [19]. Mitf is the master regulator of melanogenesis that regulates the expression of the melanogenic enzymes including Tyr, Tyrp1, and Tyrp2, as well as other pigmentation factors [20]. Thus, miR-27a-3p could repress Wnt3a to inhibit melanogenesis in mouse melanocytes.

Several studies have shown the effects of miRNAs on melanogenesis. For example, ectopic miR-218 dramatically reduced Mitf expression, suppressed Tyr activity, and induced depigmentation in murine immortalized melan-a melanocytes [21]. MiR-675 was shown to be involved in H19-stimulated melanogenesis by targeting Mitf gene [22]. MiR-203 reduces melanosome transport and promotes melanogenesis by targeting Kif5b and negative regulation of the CREB1/MITF/Rab27a pathway in melanoma [23]. MiR-434-5p mediated skin whitening and lightening *in vitro* and *in vivo* [24]. Furthermore, over-expression of miR-25 reduced Mitf, Tyr and Tyrp1 mRNA and protein abundance in cultured melanocytes [2]. In addition, miR-340 and miR-145 are known to be involved in the pigmentation process [13,14]. MiR-27a-3p has been reported to regulate multiple genes in cancer cells [25,26], as well as in fat metabolism and cell proliferation during hepatic stellate cell activation [27]. In the present study, we provided evidence supporting a role of miR-27a-3p in inhibiting melanogenesis.

While many studies have demonstrated the involvement of miRNAs in regulating melanogenesis, very few studies paid attention to the function of miRNAs in melanosome formation and transport. Therefore, future studies may focus on the role of miRNAs in the regulation of formation and transport of melanosome.

## 3. Experimental Section

### 3.1. Ethics Statement and Sample Collection

This study was carried out in strict accordance with the recommendations in the Guide for the Care and Use of Laboratory Animals of the National Institutes of Health (National Research Council (US) Committee for the Update of the Guide for the Care and Use of Laboratory Animals, 27 December 2010, ISBN: 9780309154000).

Skin samples of mouse with brown and gray coat color were collected and frozen at −80 °C for isolation of total RNA and protein. Mouse tails were collected for extraction of DNA.

### 3.2. Plasmid Cloning

Mouse pri-miR-27a was PCR amplified and TA cloned into the pUC-T vector (CWBIO, Beijing, China). The fragment (186 bp) was then subcloned into pMSCV PIG vector (Addgene, Cambridge, MA, USA) with Xho I and EcoR I restriction sites.

A partial 3' UTR (671 bp) of the mouse Wnt3a gene with the miR-27a-3p binding site was PCR amplified, TA cloned and then subcloned into pmirGL0 dual-luciferase miRNA target vector (Promega, Madison, WI, USA) with Sac I and Xba I restriction sites to generate the wild-type construct (pmirGL0-Wnt3a-wt-3' UTR). The mutant construct (pmirGL0-Wnt3a-mut-3' UTR) with mutations in the miR-27a-3p binding site of the Wnt3a 3' UTR sequence was created using the Site-Directed Gene Mutagenesis Kit (Beyotime, ShangHai, China) and specific primers containing mutated nucleotides (Table 1). Incorporation of the mutations in the plasmid was confirmed by sequencing.

**Table 1:** Primers used in this study.

Primer Name	Primer Sequence 5'–3'	Application
Wnt3a F	CAGTGCCTCGGAGATGGTG	Real time PCR
Wnt3a R	GGTTAGGTTCGCAGAAGTTGG	Real time PCR
Wnt3a 3' UTR F	CGAGCTCCGTGCCTGGGTACCTCTTTT	Luciferase reporter-wt
Wnt3a 3' UTR R	GCTCTAGAGAACGCAAAGTTCCAGGCAG	Luciferase reporter-wt
Wnt3a-3' UTR-mut F	TTCCTGGTTGGTACCACACACAACCGTCCCTCCCCCCT	Luciferase reporter-mut
Wnt3a-3' UTR-mut R	AGGGGGGAGGGACGGTTGTGTGTGGTACCAACCAGGAA	Luciferase reporter-mut
MiR-27a F	ACACTCCAGCTGGGTTCACAGTGGCTAAG	Real time PCR
Universal Primer	TGGTGTCGTGGAGTCG	Real time PCR
U 6 F	CTCGCTTCGGCAGCACA	Real time PCR
U 6 R	AACGCTTCACGAATTTGCGT	Real time PCR
MiR-27a R	CTCAACTGGTGTCGTGGAGTCCGGCAATTCAGTTGAGGCGGAACT	Real time PCR
MiR-27a F-XhoI	AAGCTCGAGCTGTCGCCAAGGATGTCTGT	PCR-Clone
MiR-27a R-EcoRI	GGAATTCAGGAGGCAGAGCAGGGTG	PCR-Clone
18S-F	GAAGGGCACCACCAGGAGT	Real time PCR
18S-R	CAGACAAATCACTCCACCAA	Real time PCR
β-Catenin	GTTGTACTGCTGGGACCCTT	Real time PCR
β-Catenin	CCCAAGCATTTTCACCAGCG	Real time PCR

The underline sequence are mutation site.

### 3.3. Cell Culture and Transfection

All melanocytes used in this study were established in the laboratory of alpaca biology, College of Animal Science and Technology, Shanxi Agricultural University, China. Melanocytes were maintained in melanocytes medium (MelM) (ScienCell Research Laboratories, Carlsbad, CA, USA). HEK 293 T cells were cultured in DMEM (Gibco, New York, NY, USA) supplemented with fetal bovine serum (10%).

The miR-27a-3p mimic, miR-27a-3p inhibitor and their negative control molecules were synthesized by QIAGEN (Hilden, Germany). Melanocytes were transfected using lipofectamine 2000 reagent (Invitrogen, Carlsbad, CA, USA). Transfection complexes were prepared according to the manufacturer’s instructions and added directly to the cells. Six hours after transfection, the medium was replaced with fresh serum-free medium (MelM) followed by incubation for an additional 48 h. Then, the cells were harvested to extract total RNA and protein.

The transfection groups included control (untransfected), miR-NC (transfected with miR-27a-3p mimic negative control), miR-27a (transfected with miR-27a-3p mimic), inhi-NC (transfected with miR-27a-3p inhibitor negative control), and miR-27a inhi (transfected with miR-27a-3p inhibitor).

### 3.4. Luciferase Assay

Twenty-four hours after seeding, cells were transfected with 150 ng of pmirGL0-Wnt3a-wt-3' UTR or pmirGL0-Wnt3a-mut-3' UTR together with 250 ng of pMSCV-pre-miR-27a or negative control. Forty-eight hours after transfection, cells were lysed using 1× Passive Lysis Buffer from the Dual Luciferase Assay kit (Promega, Madison, WI, USA) and luciferase activity was measured using GLOMAXTM 96 Microplate Luminometer (Promega, Madison, WI, USA) according to the manufacturer’s instructions. Firefly luciferase values were normalized to those of Renilla luciferase.

### 3.5. RNA Preparation and Real-Time PCR Analysis

Total RNA from 6 mouse skin samples (three gray and three brown) or melanocytes was isolated using the TRIzol reagent (Invitrogen, Carlsbad, CA, USA). The concentration of total RNA was determined using the NanoDrop 1000 spectrophotometer (NanoDrop, Wilmington, NA, USA). Synthesis of cDNA for real-time PCR analysis of miR-27a-3p expression in mouse skin and cultured melanocytes was performed using the PrimeScriptTM RT Master Mix (Perfect Real Time) kit (TAKARA, Dalian, China) and a miR-27a-3p stem loop primer according to the manufacturer’s instructions. Real-time PCR for miR-27a-3p was performed using SYBR^®^ PrimeScriptTMII RT-PCR kit (TAKARA, Dalian, China), a universal primer and a miR-27a-3p sequence-specific forward primer. All reactions were performed in triplicate on the 7500 FAST Real-Time QPCR system (Life Technologies, Grand Island, NE, USA). Quantification of miR-27a-3p transcript abundance was performed using the comparative threshold cycle (*C*_t_) method. The abundance of miR-27a-3p was normalized relative to that of U6 snRNA. Real-time PCR analysis of mRNA abundance was performed for Wnt3a and β-catenin using gene specific primers. The expression of Wnt3a and β-catenin mRNA was normalized relative to the abundance of 18S rRNA. All primer sequences are listed in Table 1.

### 3.6. Protein Extraction and Western Blot Analysis

Total protein was extracted using tissue protein extraction reagent (Boster, Wuhan, China), and the concentrations were determined using the NanoDrop 1000 spectrophotometer (NanoDrop, Wilmington, NA, USA). One hundred micrograms of protein extract per sample were resolved on 4%–10% gels by SDS-PAGE electrophoresis and transferred to nitrocellulose filter membranes (Millipore, New York, NY, USA). After blocking in 10% skimmed milk powder (Boster, Wuhan, China) at room temperature for 1 h, the membranes were washed three times using Tris-buffered saline-Tween (TBST), each for 10 min. The membranes were then incubated with the rabbit anti-Wnt3a primary antibody (1:1000 dilution (*v*/*v*), Abcam, Cambridge, MA, USA) or a rabbit anti-β-actin primary antibody (1:3000 dilution (*v*/*v*), Boster, Wuhan, China) overnight at 4 °C. The next day, the membranes were washed three times by TBST, each for 10 min, and incubated with horseradish peroxidase (HRP)-conjugated goat anti-rabbit-IgG (1:3000 (*v*/*v*), Boster, Wuhan, China) at room temperature for 1 h. Finally, the membranes were washed and a super ECL chemiluminescence plus (Boster, Wuhan, China) was used for visualization. The Image Lab software associated with the Bio-Rad system was used to scan and visualize the band intensities of Wnt3a and β-actin proteins. The level of Wnt3a protein expression was normalized relative to corresponding β-actin level in each lane.

### 3.7. Melanin Measurement

Melanocytes were washed with PBS for three times 72 h after transfection. The cells were digested with 0.25% trypsin for 8 min and centrifuged at 1000 rpm for 10 min at 4 °C. Cells were resuspended in PBS and counted using a hemocytometer. The cells were pelleted again, and 1 mL of 1 M NaOH was added followed by mixing and incubation at 37 °C for 1 h. Melanin content was then measured at 490 nm using a Multiscan Spectrum microplate reader (Thermo, Waltham, MA, USA). The melanin content was normalized relative to miR-27a group.

### 3.8. Statistical Analysis

The differences in abundance of miR-27a-3p and Wnt3a mRNA between gray and brown mouse skin, and the differences in Wnt3 and β-catenin mRNA, Wnt3a protein and melanin content in miR-NC, miR-27a, inhi-NC, miR-27a inhi and control groups (*n* = 3) were determined by analysis of variance using SPSS software (IBM, Armonk, NY, USA).

## 4. Conclusions

In conclusion, our results demonstrated that Wnt3a is a target of miR-27-3p, and miR-27a-3p can inhibit melanogenesis in melanocytes. Wnt3a could promote melanogenesis in mouse melanocytes. So miR-27-3p represses Wnt3a protein expression to inhibit melanogenesis.
